# Supplementary material for: rworkflows: automating reproducible practices for the R community
Source: Nat Commun. 2024 Jan 2;15:149. doi: 10.1038/s41467-023-44484-5 (PMC10761765; doi:10.1038/s41467-023-44484-5)
Supplement: Supplementary file 1 — Supplementary Information [file 41467_2023_44484_MOESM1_ESM.pdf]

# Supplementary Information

## Links

*act*:
https://github.com/nektos/act
Codecov:
https://codecov.io
Coveralls:
https://coveralls.io
Docker Hub:
https://hub.docker.com
GitHub Actions documentation:
https://github.com/features/actions
GitHub Pages:
https://pages.github.com
GitHub integration with Zenodo/Figshare:
https://docs.github.com/en/repositories/archiving-a-github-repository/referencing-and-citing-content
GitHub code search for R package DESCRIPTION files:
https://github.com/search?q=%2F%28%3F-i%29Package%3A%2F+path%3A%2F%28%3F-i%29%5EDESCRIPTION%24%2F&type=code
Neurogenomics Lab YouTube channel:
https://youtube.com/@NeurogenomicsLab
Wiki page on creating a Bioconductor package:
https://github.com/neurogenomics/labwiki/wiki/Creating-a-Bioconductor-package
Step-by-step guide for creating Bioconductor R packages:
https://neurogenomics.github.io/bioc_mentorship_docs/bioc-package.html
https://bioconductor.github.io/bioc_mentorship_docs/bioc-package.html
All GitHub repositories that have implemented the *rworkflows* action:
https://github.com/neurogenomics/rworkflows/network/dependents
*rworkflows::use_workflow* arguments documentation

# Tables

## Glossary.

Definitions of acronyms and terms used in this paper.

| Category | Term | Acronym | Definition | URL |
|---|---|---|---|---|
| computer science | Application Programming Interface | API | A method of interacting with a software or database programmatically. | https://docs.github.com/en/rest |
| computer science | Findable Accessible Interoperable Reusable | FAIR | A core set of principles designed to improve the impact of science. | https://www.go-fair.org/fair-principles/ |
| computer science | container | - | A self-contained emulation of a computer system within another computer system. | https://docs.docker.com/glossary/ |
| computer science | image | - | An instance of a container. | https://docs.docker.com/glossary/ |
| computer science | Continuous Integration | CI | Automated checks to software upon making changes. | https://docs.github.com/en/actions/automating-builds-and-tests/about-continuous-integration |
| computer science | Continuous Deployment | CD | Automated releases to software upon making changes. | https://docs.github.com/en/actions/automating-builds-and-tests/about-continuous-integration |
| computer science | dependency | | A software that another software depends on. | |
| computer science | Docker | - | A type of container. | https://www.docker.com/ |
| computer science | Docker Hub | - | A repository for hosting and distributing Docker containers. | https://hub.docker.com/ |
| computer science | GitHub Container Registry | GHCR | A service provided by GitHub to host and distribute container images. | https://docs.github.com/en/packages/working-with-a-github-packages-registry/working-with-the-container-registry |
| computer science | Operating System | OS | The base software that | https://docs.github.c |

| | | | | |
|---|---|---|---|---|
| | | | runs all other software on a computer. | om/en/actions/using-github-hosted-runners/about-github-hosted-runners |
| computer science | Singularity | - | A type of container. | https://docs.sylabs.io/guides/3.5/user-guide/introduction.html |
| computer science | Virtual Machine | VM | A self-contained emulation of a computer system within another computer system. | https://docs.github.com/en/actions/using-github-hosted-runners/about-github-hosted-runners |
| computer science | YAML Ain't Markup Language | yaml | A hierarchical text file format that can be used to create scripts for GHA workflows and actions. | https://docs.github.com/en/actions/using-workflows/workflow-syntax-for-github-actions |
| computer science | README | - | The primary documentation file describing a software and/or a set of files in a folder. | https://docs.github.com/en/repositories/managing-your-repositorys-settings-and-features/customizing-your-repository/about-readmes |
| GitHub | commit | - | A git command to document what changes have been made to the code and prepare them to be pushed to the remote repository. | https://github.com/git-guides |
| GitHub | GitHub Actions | GHA | A feature of GitHub that allows developers to automatically deploy continuous integration workflows. | https://github.com/features/actions |
| GitHub | GitHub Actions Marketplace | GHA Marketplace | A feature of GitHub that allows users to reuse actions developed by other users. | https://github.com/marketplace |
| GitHub | GitHub Pages | - | A feature of GitHub that allows users to host static website directly from code stored in GitHub repositories. | https://pages.github.com/ |

| | | | | |
|---|---|---|---|---|
| GitHub | pull request | - | A git command used to request integrating changes to code into a repository. | https://github.com/git-guides |
| GitHub | push | - | A git command used to send local changes in code to a remote repository. | https://github.com/git-guides |
| R function | *BiocCheck::BiocCheck()* | - | R function for checking R package standards. | http://bioconductor.org/packages/release/bioc/html/BiocCheck.html |
| R function | *rcmdcheck::rcmdcheck()* | - | R function for checking R package standards. | https://r-lib.github.io/rcmdcheck/reference |
| R function | *remotes::install_github()* | - | R function for installing R packages directly from a GitHub repository. | https://remotes.r-lib.org/reference |
| R function | *rworkflows::use_workflow()* | - | R function for creating a new *rworkflows* workflow file. | https://neurogenomics.github.io/rworkflows/reference |
| R function | *rworkflows::use_badges()* | - | R function for creating a set of badges in markdown and HTML format. | https://neurogenomics.github.io/rworkflows/reference |
| R function | *rworkflows::use_dockerfile()* | - | R function for creating a generalisable Docker receipe file (Dockerfile) which contains instructions for creating a Docker image with specified software preinstalled. | https://neurogenomics.github.io/rworkflows/reference |
| software package | *pkgdown* | - | R package to help developers generate documentation websites for their R packages. | https://pkgdown.r-lib.org/ |
| software package | *biocthis* | - | R package to help developers document and test their R packages. | https://www.bioconductor.org/packages/release/bioc/html/biocthis.html |
| software package | *act* | - | Command line software for checking GitHub Actions locally. | https://github.com/nektos/act |
| software package | *testthat* | - | R package for unit testing. | https://testthat.r-lib.org/ |

| | | | | |
|---|---|---|---|---|
| software package | *RUnit* | - | R package for unit testing. | https://cran.r-project.org/web/packages/RUnit |
| R package repository | rOpenSci | - | Dedicated R package repository for science. | https://ropensci.org/ |
| R package repository | R-Forge | - | Dedicated R package repository. | https://r-forge.r-project.org/ |
| R package repository | Bioconductor | Bioc | Dedicated R package repository for biology-related softwares and datasets. | https://bioconductor.org/ |
| R package repository | The Comprehensive R Archive Network | CRAN | Dedicated R package repository. | https://cran.r-project.org/ |
| *rworkflows* component | *rworkflows* action | - | An action hosted on the GHA marketplace. This actions is maintained on a centralised GitHub repository so that any fixes/updates can be propogated to all users of the action without having to make any changes themselves. | https://github.com/marketplace/actions/rworkflows |
| *rworkflows* component | *rworkflows* R package | - | R package with functions to set up the *rworkflows* workflow file, as well as badge creation. | https://github.com/neurogenomics/rworkflows/ |
| *rworkflows* component | *rworkflows* workflow | - | A short GHA workflow script (written in yaml format) containing only a call to the *rworkflows* action and user-supplied parameters. | https://github.com/neurogenomics/rworkflows/ |
| *rworkflows* component | *templateR* | - | A template for CRAN/Bioc-compatible R packages with autofilled documentation and vignettes. This template can easily be forked by anyone with a GitHub account so that they can start developing high-quality R packages straight away with minimal | https://github.com/neurogenomics/templateR |

| | | | | |
|---|---|---|---|---|
| | | | setup time. | |
| *rworkflows* component | *rworkflows* suite | - | Refers to all components of *rworkflows* as a whole. | https://github.com/neurogenomics/rworkflows/ |
